# Clinical and laboratory test in patients with familial amyloid polyneuropathy: differences between symptomatic patients and asymptomatic carriers

**DOI:** 10.1186/1750-1172-10-S1-P30

**Published:** 2015-11-02

**Authors:** Manuel Raya-Cruz, Juan Buades-Reines, Cristina Gallego-Lezaun, Ignacio Ferullo, Tomas Ripoll-Vera, Mercedes Uson-Martín, Hernan Andreu-Serra

**Affiliations:** 1 Son Llátzer Hospital, Internal Medicine, 07198, Palma de Mallorca, Spain; 2 Son Llátzer Hospital, Cardiology, 07198, Palma de Mallorca, Spain; 3 Son Llátzer Hospital, Neurology, 07198, Palma de Mallorca, Spain; 4 Son Llátzer Hospital, Digestive, 07198, Palma de Mallorca, Spain

## Background

Transthyretin-associated Familial Amyloid Polyneuropathy (TTR-FAP) is a frequent disease in our community caused by the deposit of abnormal transthyretin on the tissues, mainly on the nerves. The clinical affectation and laboratory test alterations depend on the clinical stage and the moment of disease diagnosis.

## Methods

A cross-sectional, observational study was performed. Medical records and laboratory test information of 20 patients: 10 symptomatic patients and 10 asymptomatic carriers.

## Results

Out of a total of 20 patients: 14 women (70 %) with a median age of 47.5 years. All of asymptomatic carriers were diagnosed for family history and 90 % of the symptomatic patients had neurologic impairment demonstrated with pathological electroneurography (NC) (p=0.016). The symptomatic patients had higher variability of blood pressure both systolic (p=0.016) and diastolic (p=0.045) and of heart rate (p<0.005). Regarding laboratory test alterations this patients presented a decrease of free T4 (p<0.005) and an increase of cystatine C (p=0.046). As for the comparison by age-at-onset in 9 (45 %) cases the diagnosis was late-onset and 11 (55 %) early-onset. Mean age was 38.55 vs 61.56 years (p<0.005). The 63.6 % of the patients less than 50 years old were diagnosed for family history and all were asymptomatic, 62.5% of them had normal NC and 9.1 % were in clinical stage II. The late-onset in comparison with the other group had a decrease of total proteins (p=0.008) and an increase of Blood urea nitrogen (p<0.005) and cystatine C (p=0.04).

## Conclusions

Symptomatic patients were diagnosed by the presence of neurologic symptoms by pathological NC, postural hypotension, and laboratory abnormalities of kidney and thyroid function. As to the comparison of age-at-onset, the early-onset has greater family history, minor number of affected organs, low neurological involvement and mild symptoms.

